# The Structure of Cognitive Abilities and Associations with Problem Behaviors in Early Adolescence: An Analysis of Baseline Data from the Adolescent Brain Cognitive Development Study

**DOI:** 10.3390/jintelligence11050090

**Published:** 2023-05-10

**Authors:** Dawn Michele Moore, Andrew R. A. Conway

**Affiliations:** 1 Division of Behavioral and Organizational Sciences, Claremont Graduate University, Claremont, CA 91711, USA; 2 Department of Psychology, New Mexico State University, Las Cruces, NM 88003, USA

**Keywords:** cognitive function, factor analysis, internalizing, externalizing

## Abstract

Using baseline data (*n* = 9875) from the Adolescent Brain Cognitive Development (ABCD) Study examining children aged 9 to 10 years, the current analyses included: (1) exploratory factor analysis (EFA) and confirmatory factor analysis (CFA) of neurocognitive measures administered during baseline collection, and (2) linear regression analyses on the Child Behavior Checklist (CBCL), controlling for demographic and socioeconomic factors. The neurocognitive tasks measured episodic memory, executive function (EF; attention), language skills, processing speed, working memory, visuospatial ability, and reasoning. The CBCL included composite scores of parent-reported internalizing, externalizing, and stress-related behavior problems. The study reported here serves as an extension of prior research using a principal components analysis (PCA) of the ABCD baseline data. We propose an alternative solution using factor analysis. Analyses revealed a three-factor structure: verbal ability (VA), executive function/processing speed (EF/PS), and working memory/episodic memory (WM/EM). These factors were significantly correlated with the CBCL scores, albeit with small effect sizes. These findings provide a novel three-factor solution to the structure of cognitive abilities measured in the ABCD Study, offering new insights into the association between cognitive function and problem behaviors in early adolescence.

## 1. Introduction

“No more a child, not yet an adult” (Brizio et al. 2015). Flanked by childhood and adulthood, adolescence is a transitional period of life marked by substantial development, increased exploration, and heightened self-consciousness (Mills et al. 2014). These considerable social and developmental changes are accompanied by enhanced cognitive functioning (Thompson et al. 2019). Psychological growth in adolescence, much like physical growth, is extremely variable, even in healthy populations. For example, in various social skills and cognitive abilities, children exhibit different overall amounts of change and patterns of growth. A primary goal of psychological science is to identify risk factors that negatively impact psychological growth in child development. The identification of risk factors is essential for future prevention and intervention efforts as well as diagnosis and treatment plans.

A major challenge for developmental research is how to best measure cognitive function in children and adolescents. An obvious methodological problem is that tasks designed for adults may not be appropriate or adaptable for children. A less obvious, more theoretical issue is that the structure of cognitive abilities may change over the course of child development. Also, depending on investigator goals, it may be preferable to focus on broad cognitive constructs, such as verbal ability or fluid reasoning, or more specific constructs, such as phonological awareness, executive function (EF), or working memory (WM).

### 1.1. ABCD Study

The primary aim of the current project is to examine the structure of cognitive abilities measured in the ABCD Study, the largest longitudinal study on brain development and adolescent health in the United States, following over 11,800 children from pre-adolescence into young adulthood (ABCD 2021). The baseline data used in the current analyses were collected from adolescents aged 9 to 11 years and their parents/guardians. The main objective of the ABCD Study “is to produce for the scientific community an informative, high-dimensional data resource, populated by assessments with strong validity and good quality” (Jernigan et al. 2018). To that end, the ABCD Study included several well-established neurocognitive and mental health measures. The neurocognitive measures are based on the NIH Toolbox Cognition Battery (Toolbox-CB), comprised of seven distinct tasks measuring five constructs: episodic memory, EF (attention), language skills, processing speed, and WM (Luciana et al. 2018). In addition to the Toolbox-CB, the ABCD Study included three other neurocognitive measures: the Rey Auditory Verbal Learning Test (RAVLT), the Little Man Task (LMT; visual spatial processing), and the WISC-V Matrix Reasoning Task (ABCD 2021).

Mental health measures included in the ABCD Study assess various behavioral outcomes associated with adolescence. The secondary aim of the current study is to examine the relationship between cognitive abilities and behavioral outcomes measured by the CBCL. Completed by parents during the baseline collection period, the CBCL assesses problem behaviors, including anxiety, depression, withdrawing, sleep/somatic issues, aggressive/destructive behaviors, internalizing/externalizing, and stress problems (Achenbach and Ruffle 2000; Barch et al. 2018).

### 1.2. Factor Structure of Cognitive Abilities

As mentioned above, the Toolbox-CB consists of seven tasks measuring five constructs. However, the results of factor analytic studies on the Toolbox-CB are somewhat mixed. For example, in an initial summary report of Toolbox-CB studies, Bauer and Zelazo (2014) reported that a series of confirmatory factor analyses (CFA) revealed five- and three-factor models for older (8- to 15-year-olds) and younger (3- to 6-year-olds) children, respectively. They also reported that the correlation between composite scores representing fluid and crystallized intelligence was higher among the younger children than among the older children, which is indicative of greater differentiation of cognitive abilities with age. In a study on 8- to 15-year-olds (*n* = 88), which is most relevant here, Mungas et al. (2014) provided evidence for a five-factor model, but suggested a slightly different set of factors than was suggested by Luciana et al. (2018): vocabulary, reading, episodic memory, WM, and EF/PS (Mungas et al. 2014). Furthermore, while the Mungas et al. five-factor model provided the best overall fit, the inter-factor correlations were extremely high, especially between vocabulary and reading (*r* = .82) and episodic memory and WM (*r* = .77). Their five-factor model also included a cross-loading between episodic memory and WM, raising further concern about discriminant validity in the measurement model. Taken together, the results of Mungas et al. suggest the plausibility of a more parsimonious three-factor model: VA, episodic memory/WM, and EF/PS.

Given the inherent challenges in determining the factor structure of cognitive abilities measured by the Toolbox-CB, some researchers have opted for data-driven approaches, such as PCA and the creation of composite scores. For example, Heaton et al. (2014) conducted a Toolbox-CB study (*n* = 268) and found strong reliability and validity for two composite scores, which they referred to as fluid cognition and crystallized cognition. More recently, and most relevant to the current project, Thompson et al. (2019) conducted a PCA on baseline data from the ABCD Study (*n* = 4093) and provided evidence for a three-component solution: general ability, learning/memory, and EF.

At first glance, the results of this initial set of studies appear to be mixed (five factors vs. two composites vs. three components). However, upon closer inspection, there is a somewhat consistent pattern (Thompson et al. 2019). For example, a comparison of the three-composite solution from Thompson et al. (general ability, learning/memory, EF) and the above mentioned three-factor solution from Mungas et al. (VA, WM/EM, EF/PS), suggests a similar factor pattern (despite different labels for the first factor, i.e., general ability vs. VA). Also, the tasks associated with the first component in Thompson et al. and the first factor in Mungas et al. are primarily measures of crystallized cognition, and the variables associated with the second and third component in Thompson et al. and second and third factor in Mungas et al. are primarily measures of fluid cognition. Again, taken together, there is evidence of a somewhat consistent pattern across studies. This is reassuring.

However, technically speaking, Thompson et al. (2019) did not identify the factor structure of cognitive abilities measured in the ABCD Study because they did not report a factor analysis. They conducted a PCA with varimax rotation. In our view, this approach is problematic for several reasons. First, PCA assumes that observed variables are measured without error and that all observed variance across tasks is “true” variance. This is not a reasonable assumption in the ABCD Study where the observed variables are scores on cognitive tasks. Factor analysis is more appropriate than PCA in this situation because factor analysis explicitly assumes that the observed variables are impacted, to some extent, by measurement error. Second, varimax rotation returns an orthogonal solution, which ignores any correlations that may exist between the factors. That is, the three components identified by Thompson et al. (general ability, learning/memory, EF) are assumed to be independent of one another. This is inappropriate because it is well-established that cognitive abilities are positively correlated (Kovacs and Conway 2016; Spearman 1904). In addition, given that orthogonal rotations are not able to effectively deal with correlated factors, an oblique rotation is more appropriate (Osborne 2015). Third, and most importantly, the factor structure of cognitive abilities implied by the PCA solution in Thompson et al. is incompatible with most contemporary theories and psychometric models of intelligence (Kovacs and Conway 2016). Specifically, the first component of their PCA solution is “general ability.” According to leading psychometric models of intelligence (e.g., the Cattell–Horn–Carroll (CHC) model, Schneider and McGrew 2012), “general ability” is associated with a higher-order general factor, which is thought to reflect domain-general variance shared across broad ability factors (and a broad range of tasks). In the Thompson et al. PCA solution, the measures that load most strongly on “general ability” are verbal tasks (e.g., oral reading, picture vocabulary). Not only is this inconsistent with contemporary models of intelligence, but it results in an incoherent structure of cognitive abilities; the first component is “general ability,” which is mainly comprised of domain-specific verbal tasks and the second component is “executive function,” which is mainly comprised of domain-general attention tasks. Finally, from a cognitive psychology perspective, it is not clear how “general ability” could be orthogonal to “executive function” or “learning/memory”.

Given these concerns, in the current study, we conducted both EFA and CFA on the ABCD Study baseline data, informed by cognitive psychology and psychometrics, to examine the factor structure of cognitive abilities. In our view, a combined cognitive/psychometric approach is essential for psychological science (Cronbach 1957). In this approach, factor analysis is conducted on measures of task performance to determine the factor solution, which explicitly implies a psychometric model and implicitly suggests a cognitive model. The validity and quality of the factor solution are based on correlational data in support of the psychometric model (model fit), experimental and theoretical evidence in support of the cognitive model, and compatibility between the two models. Importantly, the factor solution is a measurement model, whereby latent variables are thought to have causal effects on the measures of task performance. In other words, the model “explains” individual differences in cognitive function. In contrast, in a PCA, each component is a linear combination of the measures of task performance, with component scores simply being weighted averages of the measures. This means the model is “explained by” individual differences in task performance.

To preview the current results, we present evidence for a three-factor solution of the ABCD baseline data. The three factors, VA (Justice et al. 2017; Karasinski 2015; Miller et al. 2018), EF/PS (Diamond 2013; Friedman and Miyake 2017; Miyake and Friedman 2012; Miyake et al. 2000; Zelazo 2015), and WM/episodic memory (Conway and Kovacs 2013; Engle et al. 1999; Peter-Hagene et al. 2019; Tuholski et al. 2001), are all based on highly valid individual differences constructs with substantial theoretical foundations. In addition to the EFA results, we show that a three-factor CFA model provides an excellent fit to the data. Moreover, the proposed CFA model is compatible with contemporary psychometric models of intelligence, e.g., the CHC model (Schneider and McGrew 2012) and process overlap theory (Kovacs and Conway 2016).

### 1.3. Internalizing and Externalizing Problems in Adolescent Development

Internalizing problems are typically characterized by depression, anxiety, sadness, loneliness, and withdrawing (Hatoum et al. 2018; Patwardhan et al. 2021; Wang and Zhou 2019), whereas externalizing problems present as impulsivity, aggression, hyperactivity, delinquency, and anti-social behaviors (Hatoum et al. 2018; Patwardhan et al. 2021). Children exhibiting internalizing or externalizing behavior are at risk for future psychopathology, making these behaviors of utmost concern to public health officials, researchers, educators, and parents (Patwardhan et al. 2021). For this reason, it is important to determine the factors that may contribute to these behavioral outcomes, as potentially predictive associations may emerge allowing for the possibility of early interventions (Patwardhan et al. 2021).

Given that EF involves self-regulation processes associated with top–down control of cognitive, emotional, and behavioral mechanisms (Wang and Zhou 2019), it is not surprising that EF is considered to play a role in internalizing and externalizing behaviors, with prior research demonstrating that various EF tasks may be related to externalizing/internalizing behaviors throughout development (Hatoum et al. 2018). More specifically, studies have demonstrated that children with lower EF skills exhibit greater internalizing and externalizing behaviors (T. D. Nelson et al. 2018; Woltering et al. 2016), whereas children displaying higher EF may exhibit fewer of these behavioral issues (Eisenberg et al. 2009). Indeed, this inverse relationship between EF and internalizing/externalizing behaviors is an extremely robust finding. In a meta-analysis of 22 studies and 69 EF assessments, Schoemaker et al. (2013) reported all negative correlations, with correlations ranging from *r* = −.07 to *r* = −.59, and a mean correlation of *r* = −.22.

While it is clear that EF is associated with internalizing/externalizing behaviors, it is not clear whether EF is uniquely or directly associated with problem behaviors. This is because the bulk of the evidence linking EF and problem behaviors is correlational and studies often fail to measure or control for other cognitive abilities. It is well-established that EF is positively correlated with several other cognitive abilities, including VA and WM, so it is possible that the previously observed relationship between EF and problem behaviors has been confounded by other variables. In fact, several studies show that WM, when measured separately from EF, is associated with internalizing/externalizing behaviors (Schoemaker et al. 2013). In addition, there is evidence to suggest that externalizing behaviors are associated with language skills and verbal ability (J. R. Nelson et al. 2005). Thus, it is possible that children with lower cognitive abilities in general (not just EF), are more susceptible to problem behaviors.

Additionally, there continue to be concerns related to task impurity issues associated with EF measurement (Miyake and Friedman 2012; T. D. Nelson et al. 2018). Given that any individual EF (e.g., WM/updating, cognitive flexibility/shifting, inhibitory control/inhibiting) is naturally embedded within a particular task environment, non-EF processes related to that particular task environment (e.g., Stroop task color or word processing) will necessarily introduce systematic variance related to the non-EF processes (Friedman and Miyake 2017; Miyake and Friedman 2012). The systematic variance and measurement error associated with these non-EF processes complicates the ability to effectively measure the particular EF of interest.

Of course, task impurity issues can be reduced by using statistical analyses that employ a latent variable model (e.g., factor analysis or structural equation modeling) (Miyake and Friedman 2012). However, the structure of cognition appears to undergo dramatic changes between early childhood and adolescence (Brydges et al. 2014; Thompson et al. 2019). As such, considerable research has been devoted to investigating and analyzing cognitive processes, particularly EFs, to determine whether EF is unitary or diverse at various ages, and to identify when during typical development the shift from unitary to diverse EF occurs (Brydges et al. 2014; Thompson et al. 2019). For example, using longitudinal factor analyses, Brydges et al. (2014) tested 135 children twice over a period of two years (once at 8 years and again at age 10) on various shifting, WM, and inhibition tasks to evaluate whether any changes in the EF structure were evident across this age range. Study findings revealed that EF shifted from a unitary, one-factor model in early childhood (age 8), to a two-factor model by age 10 (with WM loading as a separate factor, though related to an inhibition/shifting factor). Similarly, Wiebe et al. (2011), examined 228 3-year-old children using CFA, requiring the children to complete various EF tasks associated with WM and inhibitory control. Results indicated that as young as age 3 years, EF skills seem to be unitary in nature and more representative of a singular domain-general ability. Although these findings suggest different conclusions about when exactly during childhood EF shifts from a unitary factor structure to a two- or three-factor structure, there appears to be consensus that such a shift *does* occur at some point during the transition from childhood to adolescence (Brydges et al. 2014; Miyake and Friedman 2012; Mungas et al. 2013; Thompson et al. 2019; Wiebe et al. 2011).

In a recent study using EFA and CFA, Neumann et al. (2021) examined the factor structure of the NIH Toolbox-CB (the same measures used in the ABCD Study). In this large study (*n* = 4298), 8-year-old children completed the seven subtests of the Toolbox-CB. Results revealed a three-factor solution comprised of crystallized cognition (defined as vocabulary and reading), fluid cognition I (defined as attention/inhibitory control, cognitive flexibility, and processing speed), and fluid cognition II (defined as WM and EM).

Based on these results, we expect EF and WM to emerge as distinct factors in the baseline data of the ABCD Study. This will allow us to treat EF and WM as independent predictors of internalizing/externalizing behaviors. Therefore, in the current study, the regression models predicting problem behaviors will include EF, WM, and VA. This approach will allow for a more precise estimate of the unique association between EF and internalizing/externalizing behaviors.

### 1.4. Present Study

The present study is an extension of Thompson et al. (2019). However, instead of PCA, EFA and CFA were conducted on ABCD baseline data to identify latent factors that may be related to broader traits of internalizing and externalizing symptoms in adolescents. Factor scores were extracted from the CFA model and associated with CBCL externalizing and internalizing symptoms reported by parents/guardians. The analyses controlled for demographic and socioeconomic factors. Given the large number of participants involved, the ABCD Study offers a unique opportunity to explore the factor structure of the Toolbox-CB (and other cognitive measures), as well as the relationship between the resulting factor structure and problem behaviors affecting many adolescents. With these goals in mind, the aim of the present study is to:

(a) Use a psychometrically-sound data reduction technique (i.e., factor analysis) to explore the latent factor structure of cognitive abilities as measured in the ABCD Study;
(b) Examine the relationship between these cognitive abilities and behavioral outcomes (i.e., internalizing, externalizing, and stress) as measured by the CBCL.

## 2. Method

### 2.1. ABCD Study Design and Sample

Participants included 9875 children aged 9 to 11 years (*M* = 9.91, *SD* = 0.62; 48% female) obtained from the ABCD Study baseline dataset released in 2019 (ABCD 2021). Researchers from 21 research sites around the United States are currently tracking biological and behavioral development from pre-adolescence to young adulthood, using standardized assessments of neurocognition, physical/mental health, social/emotional functions, culture/environment, and brain imaging/biospecimen collection for genetic and epigenetic analyses. Curated data from the ABCD Study are released on an annual basis through the NIMH Data Archive. Data collection and release will continue for 10 years until participants reach 19–20 years of age. The sample includes participants identifying as 54.9% White/Caucasian, 13.7% Black/African American, 18.9% Hispanic/Latinx, 2.1% Asian, and 10.4% identifying with more than one race/other races (see Table 1). ABCD Study information can be obtained at http://abcdstudy.org.

### 2.2. Measures

Neurocognitive measures used in the current analyses included the Toolbox-CB, RAVLT, WISC-V Matrix Reasoning, and the LMT. Adolescent problem behaviors were measured using the parent-rated CBCL.

#### 2.2.1. Toolbox-CB

Toolbox-CB tasks are designed to assess attention, EF, WM, processing speed, and English language vocabulary skills, by age group (HealthMeasures 2021). Tasks used in the baseline collection were intended for children aged 7–17 years. Each task yielded a raw score, an uncorrected standard score, and an age-corrected standard score (Casaletto et al. 2015; Thompson et al. 2019). Just as in Thompson et al. (2019), the current analyses used the uncorrected standard scores for each task. Participants completed tasks on an iPad, with a total administration time of around 35 min. Although English and Spanish versions of the tasks exist, all tasks in the ABCD Study used the English versions, as English proficiency was a requirement for the adolescents (not the parents/guardians) to participate in the study (Luciana et al. 2018). Below are brief descriptions of each of the Toolbox-CB tasks as well as the scoring processes used. For additional details regarding the scoring processes, please refer to the NIH Toolbox Scoring and Interpretation Guide for the iPad (NIH and Northwestern University 2021).

##### Picture Vocabulary Test (Picture Vocab)

Derived from the Peabody Picture Vocabulary Test (PPVT), Picture Vocab assesses receptive vocabulary and language (Luciana et al. 2018). Respondents are required to specify the picture that, out of four picture options on a touchscreen, best matches the meaning of a specified word. For example, the four picture options may include: (a) cupcake, (b) toy, (c) hamburger, and (d) banana. After four picture blocks appear on the screen, a pre-recorded voice says a word, such as “banana.” Respondents are instructed to touch the screen to select the picture that most closely matches the specified word (HealthMeasures 2021). The test uses computer-adaptive testing (CAT), allowing for adaptations in difficulty level based on the respondent’s level of competence, and takes 4 min to complete (HealthMeasures 2021; Luciana et al. 2018). Picture Vocab uses Item Response Theory (IRT), with a theta score calculated for each participant. The present study used the uncorrected standard score. An age-corrected standard score around 100 indicated average vocabulary ability, with a score of 70 or below indicating low language ability and a score of 130 suggesting superior ability (NIH and Northwestern University 2021).

##### Flanker Inhibitory Control and Attention Task (Flanker)

Derived from the Eriksen Flanker task and the Attention Network Task (Luciana et al. 2018; Thompson et al. 2019), Flanker measures both inhibitory control and attention, with 20 trials taking 3 min to complete (HealthMeasures 2021; NIH and Northwestern University 2021). Flanker requires participants to pay attention to the middle arrow in a group of five arrows on a touchscreen. At the bottom of the screen, one arrow is pointing left and one is pointing right. In each trial, participants must press one of the two arrows on the bottom of the touchscreen that represents the direction of the middle arrow (in the five-arrow sequence). Throughout trials, arrows surrounding the middle arrow may be congruent and/or incongruent with the middle arrow. This requires the participant to stay focused on the direction of the middle arrow and avoid being distracted by the direction of the other arrows. Scoring is based on a two-vector system using the combination of reaction time and accuracy, with each of the “vectors” ranges falling between 0 and 5, and a combined score ranging from 0 to 10. With a total of 40 possible accuracy points, the accuracy score is computed as follows: 0.125 * number of correct responses. The reaction time score is calculated using a participant’s “raw, incongruent median reaction time score, with the median reaction time values computed using only correct trials with reaction times greater than or equal to 100 milliseconds and reaction times no larger than three standard deviations away from the participant’s mean” (NIH and Northwestern University 2021, p. 7) After obtaining the reaction time scores, they are added to the accuracy scores of participants who meet the accuracy requirement of better than 80%. The accuracy score is given priority over reaction time, meaning that if accuracy levels are less than or equal to 80%, the final computed score will be equal to the accuracy score alone. However, if accuracy levels are above 80%, the final combined score will be the combination of reaction time and accuracy. The combination score is then converted to normative scores. This scoring method is also used for the Card Sort task described below (NIH and Northwestern University 2021).

##### List Sorting Working Memory Test (List Sort)

Adapted from a letter–number sequencing task developed by Gold et al. (1997), List Sort uses pictures instead of numbers and letters (Luciana et al. 2018). The task involves rapid recall and ordering of different stimuli that are presented orally and verbally. Taking 7 min to complete, participants are first shown pictures of different foods and animals (both audio recording and written text). After presentation of the stimuli, participants are asked to state the items back in order by size from the smallest to the largest item on the list. In a one-category trial, participants list items from one of the categories (animals *or* foods, called one-list). In a two-category trial, participants are asked to list foods, then animals, in order from smallest to largest within each category (i.e., foods *and* animals, called two-list). Trials become increasingly difficult, reaching a maximum number of 7 items to recall in both the one-category and two-category trials (HealthMeasures 2021; Luciana et al. 2018; NIH and Northwestern University 2021). Scoring involves adding the total number of items accurately recalled and ordered on the one-list and two-list groups, with scores ranging from 0 to 26. Final scores are converted to a nationally normed standard score (NIH and Northwestern University 2021).

##### Dimensional Change Card Sort (Card Sort)

Adapted from the Dimensional Change Card Sort (DCCS) task developed by Zelazo (2006), Card Sort measures EF and cognitive flexibility, focusing on assessing the participant’s cognitive capacity to organize, plan, and monitor their behaviors in a goal-oriented way (HealthMeasures 2021). Taking 4 min to complete, participants must focus on two objects (brown boat and white rabbit) at the bottom of a touchscreen. In each trial, a third object is shown at the top of the screen that is the same shape or color as the original two objects. Trials are divided into color game trials and shape game trials. In color game trials, participants must touch the object (i.e., boat or rabbit) that matches the *color* of the third object. In contrast, in shape game trials, participants must touch the object that matches the *shape* of the third object. Additionally, “switch” trials are used, whereby participants must change the dimension being matched. For example, following four consecutive trials matching the object’s *shape*, the participant may be asked to match the object’s *color* on the next trial and then go back to matching based on *shape*, requiring the cognitive flexibility to rapidly choose the correct stimulus (HealthMeasures 2021; Luciana et al. 2018). Based on reaction time and accuracy, Card Sort uses the same scoring method as Flanker (NIH and Northwestern University 2021).

##### Pattern Comparison Processing Speed Test (Pattern Comparison)

Derived from the Salthouse et al. (1991) Pattern Comparison Task, Pattern Comparison was designed to measure visual processing speed. The task takes 3 min to complete, requiring participants to determine if two pictures, shown side-by-side, are identical or not. Participants are asked to assess the identicalness of as many picture pairs as possible in a specified period of time (HealthMeasures 2021; Luciana et al. 2018; NIH and Northwestern University 2021). Raw scores are the number of items accurately answered in 85 s of response time, with scores ranging from 0 to 130. Final scores are converted to normative standard scores (NIH and Northwestern University 2021).

##### Picture Sequence Memory Test (Picture Sequence)

Designed to measure episodic memory, Picture Sequence assesses the cognitive processes that require acquisition, storage, and retrieval of novel information (HealthMeasures 2021). Taking 7 min to complete, participants are required to arrange a set of picture tiles on a touchscreen in the correct sequence. The tiles represent an activity or event presented in a particular sequence. After viewing the sequence, the tiles are rearranged in a scrambled sequence and participants must move the tiles into empty boxes on a touchscreen in the order in which the event/activity was originally presented. The task contains two separate trials with 6–18 picture tiles and requires accuracy, not speed (HealthMeasures 2021; Luciana et al. 2018; NIH and Northwestern University 2021). Using an IRT method, the number of neighboring pairs of tiles for trials 1 and 2 are converted to a theta score that represents the participant’s estimated episodic memory ability (NIH and Northwestern University 2021).

##### Oral Reading Recognition Task (Oral Reading)

Designed to measure reading decoding skills and other cognitive skills associated with reading, this 3 min task requires participants to read and say words and letters aloud as accurately as possible (HealthMeasures 2021). Similar to Picture Vocab, Oral Reading uses CAT technology, allowing for adaptations in difficulty level, based on the respondent’s competence level. Unlike the other tasks, this task requires input from the tester, who must score each word/letter pronunciation as either correct or incorrect (HealthMeasures 2021; Luciana et al. 2018; NIH and Northwestern University 2021). Using an IRT method, the resulting theta score represents the participant’s overall reading performance/ability (NIH and Northwestern University 2021).

#### 2.2.2. RAVLT

Designed to measure auditory learning, memory, and recognition (Luciana et al. 2018; Thompson et al. 2019), RAVLT participants must listen to and recall a list of 15 unrelated words (List A) repeated over five different trials. Following the initial five trials, participants are given another list of 15 unrelated words (List B) and must recall as many words from this second list as possible. Following this distractor list of words (List B), participants are again asked to recall as many words as possible from the original list of 15 words (List A) (Trial 6, short delay). Final recall from the original list of 15 words (List A) is conducted again after a 30 min delay to assess longer-term retention (Trial 7, long delay). During the 30 min delay, participants are engaged in other non-verbal tasks. Created for the ABCD Study, an automated version of RAVLT follows the same procedure noted above but administered via an iPad with the experimenter reading each word aloud (one word per second) and checking off recalled words from the list on an iPad (Luciana et al. 2018; Strauss et al. 2006). The current analyses used the Trial 7, long delay total correct score.

#### 2.2.3. WISC-V Matrix Reasoning

Matrix Reasoning measures fluid reasoning, part–whole spatial reasoning, visual intelligence, perceptual organization, sequencing, and attention to detail. This subtest was included in the ABCD Study because fluid reasoning may be impaired by externalizing behaviors (e.g., substance use) (Keyes et al. 2017; Luciana et al. 2018). The untimed task requires participants to view an incomplete spatial array of a series of stimuli. Participants are presented with four alternative stimuli and instructed to select the one that would complete the spatial array. There are 32 possible trials with the test ending when participants fail three consecutive trials. The total number correct across all trials (i.e., the raw score) is tabulated and converted to a standard score, with the normative standard score mean equal to 10.0 and a standard deviation of 3.00 (corresponding to an IQ score of 100). The automated version of this task was used in the ABCD Study, administered via two synchronized iPads (one for the participant and one for the experimenter) (Luciana et al. 2018).

#### 2.2.4. LMT

Developed by Acker and Acker (1982), LMT measures visuospatial and non-memory skills and assesses mental rotation abilities in varying degrees of difficulty (Luciana et al. 2018; Nixon 1995). Taking between 6–8 min, the task involves the presentation of a manikin figure holding a briefcase in one of four different positions (i.e., facing away from or toward the participant while either inverted or upright). Participants must identify the hand that is holding the briefcase (Nixon 1995), and respond as quickly and accurately as possible. In the ABCD Study, children first completed practice trials with a research assistant, then completed 32 trials on their own with the position of the figure counterbalanced across trials (Luciana et al. 2018). The current analyses used the percentage correct of all 32 presented trials.

#### 2.2.5. CBCL

The CBCL was designed to record children’s problems and competencies as reported by their parents/guardians (Achenbach 1991), evaluating 113 behavioral, emotional, and social problems of children aged 6 to 18 years. Based on the prior 6-month period, parents rated items as 0 (not true as far as you know), 1 (somewhat or sometimes true), and 2 (very true or often true) (Ivanova et al. 2007). CBCL items are associated with problems on an eight-syndrome scale: anxious/depressed, withdrawn/depressed, somatic complaints, social problems, thought problems, attention problems, rule-breaking behavior, and aggressive behavior (Mazefsky et al. 2011). The CBCL also includes a score associated with stress problems and summary scores of internalizing and externalizing behaviors, with the sum of the anxious/depressed, withdrawn/depressed, and somatic complaints scores comprising internalizing behaviors, and the sum of rule-breaking and aggressive behavior scores comprising externalizing behaviors (Brislin et al. 2021; Thompson et al. 2019). The current analyses used the stress problems score and summary scores for internalizing and externalizing behaviors.

### 2.3. Statistical Approach

The analyses were conducted in *R* (Version 4.0.0) via *RStudio* (Version 1.3.1073) using the *jmv*, *psych*, *corrplot*, *ggplot2*, and *lavaan* packages. Using ABCD baseline data, both an EFA and CFA were conducted to determine the latent structure of cognitive abilities. We randomly split the data into two datasets and conducted EFA on one dataset and then CFA on the other dataset. Additionally, linear regression models were specified to examine the associations between individual differences in the CFA factors and in the domains of problem behavior (internalizing, externalizing, stress problems). Linear regression models adjusted for demographic and socioeconomic factors, including child’s age, sex, and race/ethnicity, and parent’s marital status, education, and household income.

## 3. Results

### 3.1. Descriptive Statistics

Descriptive statistics for the 10 neurocognitive assessments and CBCL behaviors are reported in Table 2. A visual inspection of the histograms for each of the neurocognitive tasks reveals normal distributions. Further supporting this conclusion, skew for all cognitive tasks is less than +/−3.00, and kurtosis is less than +/−5.00. Correlations between the 10 neurocognitive assessments are reported in Table 3.

### 3.2. Split-Sample Exploratory Factor Analysis (n = 4938)

A split-sample EFA was conducted using principal axis factoring extraction and oblique rotation (i.e., direct oblimin). Parallel analysis was used to determine the number of factors. Results of the split-sample EFA revealed a three-factor solution (the factor pattern is reported in Table 4, with factor loadings above 0.20): (a) VA, (b) EF/PS, and (c) WM/EM. Oral Reading and Picture Vocab load strongly on Factor 1 (i.e., VA), with factor loadings above 0.60, indicating a strong association with VA. In contrast, though Matrix Reasoning, List Sort, and LMT also load on Factor 1, these loadings are below 0.40, indicating weak associations with VA. Pattern Comparison, Card Sort, and Flanker load strongly on Factor 2 (i.e., EF/PS). For Factor 3 (i.e., WM/EM), Picture Sequence loads strongly and RAVLT loads moderately. The factor correlation matrix is reported in Table 5. For comparative purposes, Table 6 presents the results of Thompson et al.’s (2019) PCA, which reveals a three-component solution (only loadings above 0.40 are included in the table).

### 3.3. Split-Sample Confirmatory Factor Analysis (n = 4937)

Based on the split-sample EFA results, a split-sample CFA model with three correlated factors was specified: VA, EF/PS, and WM/EM. In the model, each factor is associated with three manifest variables. For VA, the manifest variables are Oral Reading, Picture Vocab, and List Sort. For EF/PS, the manifest variables are Pattern Comparison, Card Sort, and Flanker. For WM/EM, the manifest variables are Picture Sequence, RAVLT, and List Sort.

The determination of model fit was based on a comparison of the following fit indices: χ^2^, comparative fit index (CFI), root-mean-square error of approximation (RMSEA), and standardized root-mean-square residual (SRMR). The criteria for an “acceptable” model fit were based on the following cutoff values, recommended by Schreiber et al. (2006): *p* > .05, CFI > 0.95, RMSEA < 0.08, SRMR < 0.08.

**Figure 1:**
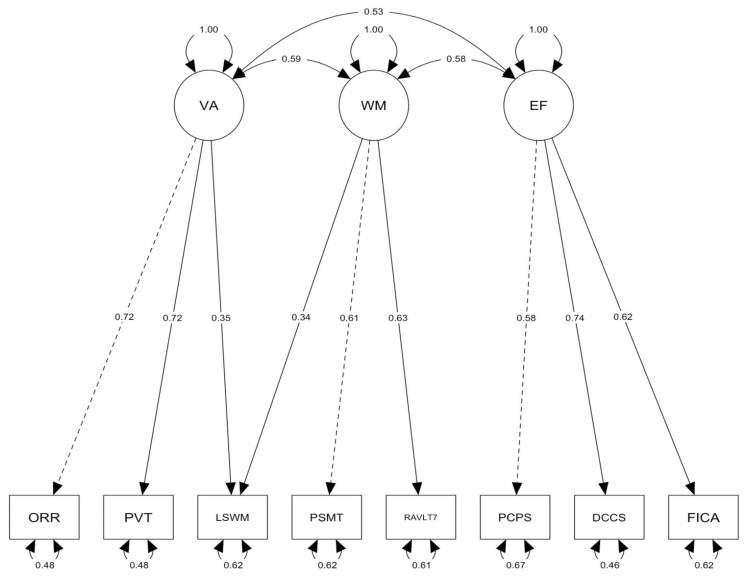
Split-sample CFA three-factor model. Task names were shortened to fit into model diagram. VA = Verbal Ability; EF = Executive Function/Processing Speed; WM = Working Memory/Episodic Memory; PVT = Picture Vocabulary; FICA = Flanker Inhibitory Control; LSWM = List Sort Working Memory; DCCS = Dimensional Change Card Sort; PCPS = Pattern Comparison Processing Speed; PSMT = Picture Sequence Memory; ORR = Oral Reading Recognition; RAVLT7 = Rey Auditory Verbal Learning Test (long delay, trial 7 only). Values represent the standardized parameter estimates. Dashed lines indicate the fixed factor loading parameters.

Overall, the results of the CFA analysis indicate a good fit. For the split-sample CFA model, χ^2^(16) = 114.04, *p* < .000, CFI = 0.99, RMSEA = 0.04, 90% CI [0.03, 0.04], and SRMR = 0.02. Although the χ^2^ statistic is significant, the other fit indices all indicated excellent fit (see Table 7 and Figure 1).

For comparison, three alternative CFA models were tested: (1) a higher-order *g* model with the same three-factor structure as our original model; (2) a bi-factor model, again, with the same three-factor structure; and (3) a two-factor model where the VA tasks load on a “crystallized cognition” factor and the remaining tasks load on a “fluid cognition” factor. The higher-order *g* model was statistically equivalent to the original model, and therefore, also demonstrated an excellent fit, with the same fit statistics [χ^2^(16) = 114.04, *p* < .000, CFI = 0.99, RMSEA = 0.04, 90% CI [0.03, 0.04], and SRMR = 0.02]. The three broad ability factors (VA, EF/PS, and WM/EM) revealed strong and consistent loadings on *g*: 0.75, 0.74, and 0.76, respectively. The bi-factor model failed to converge due to a Heywood case. The two-factor model did not provide an adequate fit to the data [χ^2^(18) = 773.93, *p* < .000, CFI = 0.91, RMSEA = 0.09, 90% CI [0.09, 0.10], and SRMR = 0.06], and a model comparison test confirmed that our three-factor solution fit the data better than the two-factor solution [Δχ^2^(2) = 659.89, *p* < .001].

As mentioned, the correlated three-factor model was statistically equivalent to the higher-order *g* model, but we choose the model without *g* because we prefer to focus on the broad cognitive ability factors (VA, EF/PS, WM/EM) and because the model without *g* is more consistent with our own theoretical perspective, which is based on process overlap theory (POT, Kovacs and Conway 2016). According to POT, *g* is a statistical index of overall cognitive ability but does not reflect an underlying psychological trait such as general intelligence. That said, we realize that other researchers may have a different theoretical perspective and prefer the higher-order *g* model, so we have reported the results of the model here and we include the code to fit the model in our R scripts, which are available online via Open Science Framework (Moore 2023).

### 3.4. Associations between Factor Scores and CBCL Measures

Following the split-sample CFA, linear regression models were used to evaluate the relationship between the three factors (i.e., VA, WM/EM, and EF/PS) and CBCL outcomes (i.e., internalizing, externalizing, and stress problems). Table 8 illustrates the correlations between the split-sample CFA factor scores and the CBCL outcomes. Table 9 shows the results of the regression analyses. For internalizing behavior, the overall model [*F*(3, 4933) = 8.70, *p* < .000, *R*^2^ = 0.005] explains 0.5% of the variance. VA, EF/PS, and WM/EM are all significant predictors of internalizing behavior; notably, the relationship between VA and internalizing is *positive* (however, the effect size is small, β = 0.079). For externalizing behavior, the overall model [*F*(3, 4933) = 50.33, *p* < .000, *R^2^* = 0.029] explains 2.9% of the variance. Both EF/PS and WM/EM are significant predictors of externalizing behavior. For stress problems behavior, the overall model [*F*(3, 4933) = 37.28, *p* < .000, *R*^2^ = 0.022] explains 2.2% of the variance. Both EF/PS and WM/EM are significant predictors of stress problems behavior. Taken together, these results suggest that there are significant associations, albeit with small effect sizes, between various cognitive abilities and the CBCL outcomes, with WM/EM and EF/PS as slightly stronger and more consistent predictors of problem behaviors than VA.

### 3.5. Association with CBCL Problem Behaviors Adjusting for Demographic Variables

Finally, the split-sample CFA factor scores were included as independent variables in linear regression models with the three CBCL outcomes, controlling for socioeconomic and demographic variables. As indicated in Table 10, the results are consistent with the previous regression results. VA is a significant *positive* predictor of internalizing behavior. Lower EF/PS and lower WM/EM predict higher levels of externalizing, internalizing, and stress problems. The change in adjusted R-squared from the baseline model (demographics) to the full model (baseline model plus all three component scores) is as follows: Externalizing: = 2.40%, Internalizing: = 0.91%, and Stress Problems = 2.35%.

## 4. Discussion

The goals of the current study were to use a psychometrically-sound data reduction technique (i.e., factor analysis) to establish the latent factor structure of cognitive abilities as measured in the ABCD Study. Further, we examined the relationship between these cognitive abilities and various problem behaviors (i.e., internalizing, externalizing, and stress) associated with adolescence as measured by the CBCL. Given the breadth and depth of the ABCD Study in terms of its diverse population of participants and the quantity/quality of available neurocognitive measures, the Study provided a unique opportunity to further explore the factor structure of the Toolbox-CB, as well as other cognitive measures (Akshoomoff et al. 2018; Mungas et al. 2013, 2014; Neumann et al. 2021).

The present study extended the analyses of Thompson et al. (2019). However, instead of a PCA, we used both exploratory and confirmatory approaches by conducting an EFA and CFA on the baseline data from the ABCD Study. There were two main findings. First, results of the EFA indicated that a three-factor solution was the best model for the 10 neurocognitive measures, yielding the following theoretically grounded constructs: (a) VA, (b) EF/PS, and (c) WM/EM. This solution was supported by our three-factor CFA model, which provided an excellent fit to the data. This factor structure is consistent with the Toolbox-CB factor structure identified by Neumann et al. (2021) [i.e., (1) reading/vocabulary, (2) attention/inhibitory control, processing speed, and cognitive flexibility, and (3) working/episodic memory]. Second, after controlling for relevant demographic factors, results of the regression models suggest that EF/PS and WM/EM are negatively associated with all three child behavior problems (i.e., internalizing, externalizing, and stress problems), while VA is positively associated with internalizing behaviors. Overall, the results suggest that EF is not uniquely associated with behavior problems in early adolescence. All three cognitive abilities (VA, EF/PS, WM/EM) were negatively correlated with externalizing and stress (see Table 8), and both WM/EM and EF/PS were significant predictors of externalizing, internalizing, and stress (see Table 9 and Table 10). That said, the effects here are small, so we are hesitant to say too much about the relative contribution of different cognitive abilities. Still, this pattern of results is intriguing, because it suggests that EF may not be as critical as previously thought.

The primary goal of the ABCD Study “is to produce for the scientific community an informative, high-dimensional data resource, populated by assessments with strong validity and good quality” (Jernigan et al. 2018). For assessments to possess strong validity and good quality, they must first be identifiable as tasks that are accurately measuring a priori constructs. To that end, one of the main objectives of the current study was to identify a factor structure of the neurocognitive tasks that would support the ABCD Study’s overall initiative, providing the psychometrically-appropriate balance between validity and quality. Our contribution to this important initiative is a three-factor solution that provides meaningful, theoretically supported cognitive constructs (i.e., VA, EF/PS, WM/EM). The importance of meaningful constructs cannot be overstated, as this is a cornerstone of good quality assessments that allow for other researchers to further explore relationships between these cognitive constructs and the various outcomes being measured by the ABCD Study. A unified, well-grounded factor structure of the ABCD neurocognitive battery is needed, as there are currently several divergent factor solutions proposed, interfering with the ability to produce robust psychological research studies. For example, models using PCA tend to ignore the inherent measurement error associated with psychological assessments (Thompson et al. 2019), a problem that is not characteristic of a factor analysis approach. Finally, composite measures reduce precision and decrease transparency (Heaton et al. 2014), making it difficult to distinguish between individual constructs, which further complicates the development of effective interventions. For these reasons, a three-factor solution appears to provide a psychometrically-appropriate balance, ensuring strong validity and high quality in theoretically solid constructs.

That said, it is important to acknowledge that the current three-factor solution is not “perfect.” As mentioned in the introduction, there is a great deal of concern with task impurity issues associated with the measurement of EF. To be clear, no cognitive task is process-pure and there will always be room for disagreement about the cognitive interpretation of factors (Kovacs and Conway 2016). For example, with respect to the current solution, the extent to which tasks such as Flanker and Card Sort successfully isolate EF from processing speed is still an open question (Frischkorn et al. 2019; Rey-Mermet et al. 2019). Likewise, the extent to which tasks such as Picture Sequence and RAVLT isolate WM from episodic memory is still a matter of debate (Baddeley 2000; Beukers et al. 2021; Unsworth and Engle 2007). Clearly, more psychometric research aimed at disentangling the contributions of EF, WM, EM, and PS to individual differences in cognitive task performance is sorely needed.

Finally, one surprising outcome in the current study is the positive relationship between VA and internalizing behavior. One the one hand, this result is fascinating because higher scores on measures of cognitive ability are rarely associated with negative life outcomes (Karpinski et al. 2018). On the other hand, the effect size observed in the current study is extremely small (β = 0.079), so we are hesitant to make too much of this finding. Nevertheless, this pattern of results is intriguing, and we will be interested to see how the relationship between various cognitive abilities and internalizing behavior unfolds in future waves of the ABCD Study.

The importance of the current study’s results is bolstered by our use of a psychometrically-sound data reduction technique (i.e., factor analysis). Although our three-factor solution is similar to the outcome of Thompson et al.’s (2019) three-component PCA solution, our use of EFA and CFA is more appropriate given the inherent measurement error associated with psychological tasks. It is well-understood throughout the psychometric literature that measurement error that is not effectively addressed using appropriate statistical methods can lead to incorrect interpretations that can inhibit the advancement of cumulative knowledge (Liu and Salvendy 2009; Schmidt and Hunter 1996). Further, although it is not possible to completely avoid measurement error, reducing this type of error strengthens the ratio between the observed variance and the true variance, improving the likelihood that the specified tasks will produce similar results over recurrent use (Knekta et al. 2019). To that end, the three-factor solution identified by our EFA and CFA provides a valid factor structure of the cognition tasks used in the ABCD Study that can be used by researchers examining future datasets.

## 5. Conclusions

In conclusion, the current study offers a novel three-factor solution to the structure of cognitive abilities measured in the ABCD Study and offers new insights into the relationship between cognitive function and problem behaviors in early adolescence. It is our sincere hope that this solution, and the factor scores derived from the current work, will be helpful to other investigators and the ABCD Study in general.

## Figures and Tables

**Table 1:** Sociodemographic characteristics of participants at baseline.

Baseline Characteristics Category ^1^	Baseline Characteristics Subcategory	*n*	%
Sex	Female	4744	48.0
	Male ^2^	5131	52.0
Race/ethnicity	White	5422	54.9
	Black	1352	13.7
	Hispanic ^2^	1873	18.9
	Asian	203	2.1
	Other	1025	10.4
Highest parental education	<HS diploma ^2^	505	5.1
	HS diploma/GED	944	9.6
	Some college	2897	29.3
	Bachelor	2894	29.3
	Post-graduate degree	2635	26.7
Parent marital status	Married	6886	69.7
	Not married ^2^	2619	26.5
	Separated	370	3.8
Household income ^3^	<50 K ^2^	2856	28.9
	≥50 K and <100 K	2811	28.5
	≥100 K	4208	42.6
Sibling status (family)	Single ^2^	6728	68.1
	Sibling	1313	13.3
	Twin	1807	18.3
	Triplet	27	0.3
Site	Site01	275	2.8
	Site02	489	5.0
	Site03	486	4.9
	Site04	605	6.1
	Site05	314	3.2
	Site06	510	5.2
	Site07	276	2.8
	Site08	303	3.1
	Site09	319	3.2
	Site10	579	5.9
	Site11	395	4.0
	Site12	501	5.1
	Site13	645	6.5
	Site14	565	5.7
	Site15	338	3.4
	Site16	934	9.4
	Site17	491	5.0
	Site18	343	3.5
	Site19	436	4.4
	Site20	606	6.1
	Site21	438	4.4
	Site22	27	0.3

^1^ *n* = 9875 for each demographic category. Participants were on average 10 years old, with baseline collection ages ranging from 9 to 11 years. ^2^ Indicates the dummy coding reference group for each variable. ^3^ Income currency = USD.

**Table 2:** Descriptive statistics of neurocognitive assessments and CBCL behaviors.

Assessments/Behaviors ^1^	*M*	*SD*	Range	Skew	Kurtosis
Picture Vocab	84.8	8.0	29–119	0.15	0.65
Flanker	94.3	8.9	54–116	−1.00	1.57
List Sort	97.1	11.9	36–136	−0.55	0.91
Card Sort	92.8	9.4	50–120	−0.82	2.21
Pattern Comparison	88.2	14.5	30–140	−0.20	−0.09
Picture Sequence	103.0	12.0	76–136	0.25	−0.40
Oral Reading	91.1	6.8	63–119	0.09	1.48
RAVLT	9.3	3.2	0–15	−0.41	−0.11
Matrix Reasoning	10.0	3.0	1–19	0.04	0.20
LMT	0.6	0.2	0–1	0.22	−0.40
INT	5.0	5.5	0–51	1.93	5.02
EXT	4.4	5.8	0–49	2.34	7.24
Stress	2.9	3.3	0–24	1.79	3.77

^1^ Picture Vocab = Picture Vocabulary; Flanker = Flanker Inhibitory Control; List Sort = List Sort Working Memory; Card Sort = Dimensional Change Card Sort; Pattern Comparison = Pattern Comparison Processing Speed; Picture Sequence = Picture Sequence Memory; Oral Reading = Oral Reading Recognition; RAVLT = Rey Auditory Verbal Learning Test (long delay, trial 7 only); Matrix Reasoning = WISC-V Matrix Reasoning; LMT = Little Man Task; INT = CBCL Internalizing score; EXT = CBCL Externalizing score; stress = CBCL Stress Problems score.

**Table 3:** Correlations for neurocognitive assessments ^1^.

Assessments ^2^	1	2	3	4	5	6	7	8	9	10
Picture Vocab	-									
Flanker	0.25	-								
List Sort	0.41	0.29	-							
Card Sort	0.29	0.44	0.32	-						
Pattern Comparison	0.19	0.38	0.21	0.42	-					
Picture Sequence	0.24	0.20	0.34	0.27	0.20	-				
Oral Reading	0.53	0.27	0.40	0.28	0.19	0.23	-			
RAVLT	0.30	0.21	0.34	0.27	0.17	0.41	0.31	-		
Matrix Reasoning	0.35	0.22	0.36	0.26	0.12	0.27	0.35	0.29	-	
LMT	0.27	0.24	0.28	0.26	0.23	0.22	0.34	0.23	0.27	-

^1^ All correlations are significant (*p* < .001). ^2^ Picture Vocab = Picture Vocabulary; Flanker = Flanker Inhibitory Control; List Sort = List Sort Working Memory; Card Sort = Dimensional Change Card Sort; Pattern Comparison = Pattern Comparison Processing Speed; Picture Sequence = Picture Sequence Memory; Oral Reading = Oral Reading Recognition; RAVLT = Rey Auditory Verbal Learning Test (long delay, trial 7 only); Matrix Reasoning = WISC-V Matrix Reasoning; LMT = Little Man Task.

**Table 4:** Split-sample EFA oblimin-rotated loadings for three-factor model ^1^.

		Factor Loadings ^3^			
Factors ^2^	Neurocognitive Tasks ^2^	1	2	3	Communality
Factor 1: VA	Oral Reading	**0.76**			0.56
	Picture Vocab	**0.69**			0.50
	Matrix Reasoning	0.37		0.25	0.30
	List Sort	0.37		0.28	0.39
	LMT	0.25			0.23
Factor 2: EF/PS	Pattern Comparison		**0.67**		0.39
	Card Sort		**0.65**		0.48
	Flanker		**0.58**		0.38
Factor 3: WM/EM	Picture Sequence			**0.71**	0.48
	RAVLT			**0.52**	0.39
Eigenvalue		1.65	1.35	1.11	
Percent of total variance		16%	14%	11%	
Total variance				41%	

^1^ *n* = 4938. ^2^ VA = Verbal Ability; EF/PS = Executive Function/Processing Speed; WM/EM = Working Memory/Episodic Memory; Picture Vocab = Picture Vocabulary; Flanker = Flanker Inhibitory Control; List Sort = List Sort Working Memory; Card Sort = Dimensional Change Card Sort; Pattern Comparison = Pattern Comparison Processing Speed; Picture Sequence = Picture Sequence Memory; Oral Reading = Oral Reading Recognition; RAVLT = Rey Auditory Verbal Learning Test (long delay, trial 7 only); Matrix Reasoning = WISC-V Matrix Reasoning; LMT = Little Man Task. For Toolbox-CB measures (Picture Vocab, Flanker, List Sort, Card Sort, Pattern Comparison, Picture Sequence, Oral Reading), uncorrected scores were used in the analyses. For RAVLT, the total correct scores for trial 7, long delay were used in the analyses. For Little Man Task, percentage correct of all 32 presented trials was used in the analyses. For WISC-V Matrix Reasoning, the total scaled score was used in the analyses. ^3^ Loadings above 0.40 are bolded.

**Table 5:** Split-sample EFA correlation matrix for three-factor model.

Factors ^1^	1	2	3
VA (Factor 1)	–		
EP/PS (Factor 2)	0.51	–	
WM/EM (Factor 3)	0.52	0.49	–

^1^ VA = Verbal Ability; EF/PS = Executive Function/Processing Speed; WM/EM = Working Memory/Episodic Memory.

**Table 6:** **Thompson et al. (2019)** varimax-rotated loadings for three-component model ^1,4^.

		Loadings ^3^		
Components ^2^	Neurocognitive Tasks ^2^	1	2	3
Component 1:	Oral Reading	0.82		
General Ability	Picture Vocab	0.75		
	LMT	0.50		
	List Sort	0.47		0.49
Component 2:	Pattern Comparison		0.81	
Executive Function	Card Sort		0.71	
	Flanker		0.71	
Component 3:	Picture Sequence			0.87
Learning/Memory	RAVLT			0.71

^1^ *n* = 4093. ^2^ Picture Vocab = Picture Vocabulary; Flanker = Flanker Inhibitory Control; List Sort = List Sort Working Memory; Card Sort = Dimensional Change Card Sort; Pattern Comparison = Pattern Comparison Processing Speed; Picture Sequence = Picture Sequence Memory; Oral Reading = Oral Reading Recognition; RAVLT = Rey Auditory Verbal Learning Test; Matrix Reasoning = WISC-V Matrix Reasoning; LMT = Little Man Task. For Toolbox-CB measures (Picture Vocab, Flanker, List Sort, Card Sort, Pattern Comparison, Picture Sequence, Oral Reading), uncorrected scores were used in the analyses. For RAVLT, the total correct scores were used in the analyses. For Little Man Task, percent correct was used in the analyses. ^3^ Only loadings above 0.40 are provided in the table above. ^4^ From “The Structure of Cognition in 9 and 10 Year-Old Children and Associations with Problem Behaviors: Findings from the ABCD Study’s Baseline Neurocognitive Battery,” by Thompson et al. (2019, p. 6). Copyright 2018 by Elsevier Ltd.

**Table 7:** Split-sample CFA standardized coefficients and associated data ^1^.

Latent Variable ^2^	Standardized Estimate	Coefficient	Standard Error	*z*-Value	*p*-Value
**VA**					
Oral Reading	0.724	1.000			
Picture Vocab	0.719	1.165	0.035	33.167	.000
List Sort ^3^	0.351	0.858	0.059	14.452	.000
**EF/PS**					
Pattern Comparison	0.576	1.000			
Card Sort	0.738	0.827	0.028	30.068	.000
Flanker	0.618	0.668	0.023	28.976	.000
**WM/EM**					
Picture Sequence	0.613	1.000			
RAVLT	0.628	0.274	0.010	26.106	.000
List Sort ^3^	0.338	0.565	0.044	12.836	.000

^1^ *n* = 4937. ^2^ VA = Verbal Ability; EF/PS = Executive Function/Processing Speed; WM/EM = Working Memory/Episodic Memory; Picture Vocab = Picture Vocabulary; Flanker = Flanker Inhibitory Control; List Sort = List Sort Working Memory; Card Sort = Dimensional Change Card Sort; Pattern Comparison = Pattern Comparison Processing Speed; Picture Sequence = Picture Sequence Memory; Oral Reading = Oral Reading Recognition; RAVLT = Rey Auditory Verbal Learning Test (long delay, trial 7 only); Matrix Reasoning = WISC-V Matrix Reasoning; LMT = Little Man Task. For Toolbox-CB measures (Picture Vocab, Flanker, List Sort, Card Sort, Pattern Comparison, Picture Sequence, Oral Reading), uncorrected scores were used in the analyses. For RAVLT, the total correct scores for trial 7, long delay were used in the analyses. For Little Man Task, percentage correct of all 32 presented trials was used in the analyses. For WISC-V Matrix Reasoning, the total scaled score was used in the analyses. ^3^ Based on the results of the EFA (see Table 4), the List Sort task was included as a manifest variable for both the VA and WM/EM latent variables.

**Table 8:** Spearman and Pearson correlations among split-sample CFA factor scores and CBCL outcomes.

Assessments ^1^	Method	1	2	3	4	5	6
VA (Factor 1)	Spearman’s rho	-					
	Pearson’s r	-					
EF/PS (Factor 2)	Spearman’s rho	0.68	-				
	Pearson’s r	0.70	-				
WM/EM (Factor 3)	Spearman’s rho	0.75	0.70	-			
	Pearson’s r	0.78	0.73	-			
EXT	Spearman’s rho	−0.12	−0.12	−0.14	-		
	Pearson’s r	−0.15	−0.14	−0.16	-		
INT	Spearman’s rho	−0.00	−0.03	−0.03	0.53	-	
	Pearson’s r	−0.02	−0.05	−0.05	0.55	-	
Stress	Spearman’s rho	−0.10	−0.11	−0.12	0.73	0.75	-
	Pearson’s r	−0.11	−0.12	−0.13	0.73	0.83	-

^1^ VA = Verbal Ability; EF/PS = Executive Function/Processing Speed; WM/EM = Working Memory/Episodic Memory; INT = CBCL Internalizing score; EXT = CBCL Externalizing score; Stress = CBCL Stress Problems score.

**Table 9:** Regression of CBCL outcomes on split-sample CFA factor scores ^1^.

Variable ^2^	Internalizing		Externalizing		Stress Problems	
	Estimate	*SE*	Estimate	*SE*	Estimate	*SE*
VA (Factor 1)	0.079 ***	0.023	−0.043	0.022	0.030	0.023
EF/PS (Factor 2)	−0.058 **	0.022	−0.050 *	0.021	−0.079 ***	0.021
WM/EM (Factor 3)	−0.064 *	0.025	−0.096 ***	0.025	−0.105 ***	0.025
*R* ^2^	0.005		0.030		0.022	

^1^ Regression analyses used standardized scores. ^2^ VA = Verbal Ability; EF/PS = Executive Function/Processing Speed; WM/EM = Working Memory/Episodic Memory. * *p* < .05; ** *p* < .01; *** *p* < .001.

**Table 10:** Regression of CBCL outcomes on split-sample CFA factor scores, adjusting for demographic variables ^1^.

	Externalizing			Internalizing			Stress Problems		
Variable ^2,3^	Coef	*SE*	*p*-Value	Coef	*SE*	*p*-Value	Coef	*SE*	*p*-Value
Intercept	0.543	0.07	.000 ***	0.515	0.07	.000 ***	0.501	0.07	.000 ***
VA (Factor 1)	−0.026	0.02	.251	0.089	0.02	.000 ***	0.035	0.02	.131
EF/PS (Factor 2)	−0.049	0.02	.020 *	−0.059	0.02	.006 **	−0.078	0.02	.000 ***
WM/EM (Factor 3)	−0.085	0.02	.001 ***	−0.054	0.03	.030 *	−0.095	0.02	.000 ***
Age	0.007	0.01	.593	0.005	0.01	.750	−0.001	0.01	.968
Female	−0.015	0.03	.595	0.019	0.03	.504	0.019	0.03	.504
White	0.038	0.04	.361	0.052	0.04	.216	0.049	0.04	.232
Black	−0.039	0.05	.452	−0.059	0.05	.256	−0.094	0.05	.067
Asian	0.033	0.11	.756	0.235	0.11	.029 *	0.168	0.11	.117
Other	−0.067	0.06	.240	−0.003	0.06	.965	0.023	0.06	.688
HS Diploma/GED	−0.475	0.09	.000 ***	−0.465	0.09	.000 ***	−0.481	0.09	.000 ***
Some College	−0.461	0.08	.000 ***	−0.491	0.08	.000 ***	−0.481	0.08	.000 ***
Bachelor	−0.569	0.08	.000 ***	−0.529	0.08	.000 ***	−0.496	0.08	.000 ***
Post-Graduate	−0.457	0.08	.000 ***	−0.501	0.08	.000 ***	−0.471	0.08	.000 ***
Married	−0.022	0.04	.542	0.001	0.04	.979	−0.004	0.04	.909
Separated	0.025	0.08	.752	−0.042	0.08	.597	−0.008	0.08	.917
≥50 K and <100 K	−0.181	0.04	.000 ***	−0.178	0.04	.000 ***	−0.170	0.04	.000 ***
≥100 K	−0.024	0.04	.544	−0.049	0.04	.231	−0.050	0.04	.216

^1^ Regression analyses used standardized scores. ^2^ VA = Verbal Ability; EF/PS = Executive Function/Processing Speed; WM/EM = Working Memory/Episodic Memory. ^3^ Reference groups for dummy-coded variables: Sex = Male; Race/ethnicity = Hispanic; Highest parental education < HS Diploma; Parental marital status = Not Married; Household income < 50 K (income currency = USD). * *p* < .05; ** *p* < .01; *** *p* < .001.

## Data Availability

To access the ABCD Data Repository, please visit the National Institute of Mental Health Data Archive (NDA): https://nda.nih.gov/abcd/.

## References

[B1-jintelligence-11-00090] Achenbach Thomas M. (1991). Manual for the Child Behavior Checklist/4-18 and 1991 Profile.

[B2-jintelligence-11-00090] Achenbach Thomas M., Ruffle Thomas M. (2000). The Child Behavior Checklist and related forms for assessing behavioral/emotional problems and competencies. Pediatrics in Review.

[B3-jintelligence-11-00090] Acker William L., Acker Clare (1982). Bexley Maudsley Automated Processing Screening and Bexley Maudsley Category Sorting Test: Manual.

[B4-jintelligence-11-00090] (2021). Adolescent Brain Cognitive Development Study (ABCD). https://abcdstudy.org/.

[B5-jintelligence-11-00090] Akshoomoff Natacha, Brown Timothy T., Bakeman Roger, Hagler Donald J., on behalf of the Pediatric Imaging, Neurocognition, and Genetics Study (2018). Developmental differentiation of executive functions on the NIH Toolbox Cognition Battery. Neuropsychology.

[B6-jintelligence-11-00090] Baddeley Alan (2000). The episodic buffer: A new component of working memory?. Trends in Cognitive Sciences.

[B7-jintelligence-11-00090] Barch Deanna M., Albaugh Matthew D., Avenevoli Shelli, Chang Linda, Clark Duncan B., Glantz Meyer D., Hudziak James J., Jernigan Terry L., Tapert Susan F., Yurgelun-Todd Debbie (2018). Demographic, physical and mental health assessments in the Adolescent Brain and Cognitive Development Study: Rationale and description. Developmental Cognitive Neuroscience.

[B8-jintelligence-11-00090] Bauer Patricia J., Zelazo Philip David (2014). The National Institutes of Health Toolbox for the assessment of neurological and behavioral function: A tool for developmental science. Child Development Perspectives.

[B9-jintelligence-11-00090] Beukers Andre O., Buschman Timothy J., Cohen Jonathan D., Norman Kenneth A. (2021). Is activity silent working memory simply episodic memory?. Trends in Cognitive Sciences.

[B10-jintelligence-11-00090] Brislin Sarah J., Martz Meghan E., Joshi Sonalee, Duval Elizabeth R., Gard Arianna, Clark D. Angus, Hyde Luke W., Hicks Brian M., Taxali Aman, Angstadt Mike (2021). Differentiated nomological networks of internalizing, externalizing, and the general factor of psychopathology (‘*p* factor’) in emerging adolescence in the ABCD Study. Psychological Medicine.

[B11-jintelligence-11-00090] Brizio Adelina, Gabbatore Ilaria, Tirassa Maurizio, Bosco Francesca M. (2015). “No more a child, not yet an adult”: Studying social cognition in adolescence. Frontiers in Psychology.

[B12-jintelligence-11-00090] Brydges Christopher R., Fox Allison M., Reid Corinne L., Anderson Mike (2014). The differentiation of executive functions in middle and late childhood: A longitudinal latent-variable analysis. Intelligence.

[B13-jintelligence-11-00090] Casaletto Kaitlin B., Umlauf Anya, Beaumont Jennifer, Gershon Richard, Slotkin Jerry, Akshoomoff Natacha, Heaton Robert K. (2015). Demographically corrected normative standards for the English version of the NIH Toolbox Cognition Battery. Journal of the International Neuropsychological Society.

[B14-jintelligence-11-00090] Conway Andrew R. A., Kovacs Kristof, Ross Brian H. (2013). Individual differences in intelligence and working memory: A review of latent variable models. The Psychology of Learning and Motivation.

[B15-jintelligence-11-00090] Cronbach Lee J. (1957). The two disciplines of scientific psychology. American Psychologist.

[B16-jintelligence-11-00090] Diamond Adele (2013). Executive functions. Annual Review of Psychology.

[B17-jintelligence-11-00090] Eisenberg Nancy, Valiente Carlos, Spinrad Tracy L., Liew Jeffrey, Zhou Qing, Losoya Sandra H., Reiser Mark, Cumberland Amanda (2009). Longitudinal relations of children’s effortful control, impulsivity, and negative emotionality to their externalizing, internalizing, and co-occurring behavior problems. Developmental Psychology.

[B18-jintelligence-11-00090] Engle Randall W., Tuholski Stephen W., Laughlin James E., Conway Andrew R. A. (1999). Working memory, short-term memory, and general fluid intelligence: A latent-variable approach. Journal of Experimental Psychology: General.

[B19-jintelligence-11-00090] Friedman Naomi P., Miyake Akira (2017). Unity and diversity of executive functions: Individual differences as a window on cognitive structure. Cortex.

[B20-jintelligence-11-00090] Frischkorn Gidon T., Schubert Anna-Lena, Hagemann Dirk (2019). Processing speed, working memory, and executive functions: Independent or inter-related predictors of general intelligence. Intelligence.

[B21-jintelligence-11-00090] Gold James M., Carpenter Constance, Randolph Christopher, Goldberg Terry E., Weinberger Daniel R. (1997). Auditory working memory and Wisconsin Card Sorting Test performance in schizophrenia. Archives of General Psychiatry.

[B22-jintelligence-11-00090] Hatoum Alexander S., Rhee Soo Hyun, Corley Robin P., Hewitt John K., Friedman Naomi P. (2018). Do executive functions explain the covariance between internalizing and externalizing behaviors?. Development and Psychopathology.

[B23-jintelligence-11-00090] HealthMeasures (Northwestern University) (2021). Cognition Measures: NIH Toolbox Cognition Batteries. https://www.healthmeasures.net/explore-measurement-systems/nih-toolbox/intro-to-nih-toolbox/cognition.

[B24-jintelligence-11-00090] Heaton Robert K., Akshoomoff Natacha, Tulsky David, Mungas Dan, Weintraub Sandra, Dikmen Sureyya, Beaumont Jennifer, Casaletto Kaitlin B., Conway Kevin, Slotkin Jerry (2014). Reliability and validity of composite scores from the NIH Toolbox Cognition Battery in adults. Journal of the International Neuropsychological Society.

[B25-jintelligence-11-00090] Ivanova Masha Y., Achenbach Thomas M., Dumenci Levent, Rescorla Leslie A., Almqvist Fredrik, Weintraub Sheila, Bilenberg Niels, Bird Hector, Chen Wei J., Dobrean Anca (2007). Testing the 8-syndrome structure of the Child Behavior Checklist in 30 societies. Journal of Clinical Child and Adolescent Psychology.

[B26-jintelligence-11-00090] Jernigan Terry L., Brown Sandra A., ABCD Consortium Coordinators (2018). Introduction. Developmental Cognitive Neuroscience.

[B27-jintelligence-11-00090] Justice Laura M., Jiang Hui, Logan Jessica A., Schmitt Mary Beth (2017). Predictors of language gains among school-age children with language impairment in the public schools. Journal of Speech, Language, and Hearing Research.

[B28-jintelligence-11-00090] Karasinski Courtney (2015). Language ability, executive functioning and behaviour in school-age children. International Journal of Language & Communication Disorders.

[B29-jintelligence-11-00090] Karpinski Ruth I., Kolb Audrey M. Kinase, Tetreault Nicole A., Borowski Thomas B. (2018). High intelligence: A risk factor for psychological and physiological overexcitabilities. Intelligence.

[B30-jintelligence-11-00090] Keyes Katherine M., Platt Jonathan, Kaufman Alan S., McLaughlin Katie A. (2017). Fluid intelligence and psychiatric disorders in a population representative sample of US adolescents. JAMA Psychiatry.

[B31-jintelligence-11-00090] Knekta Eva, Runyon Christopher, Eddy Sarah (2019). One size doesn’t fit all: Using factor analysis to gather validity evidence when using surveys in your research. CBE Life Sciences Education.

[B32-jintelligence-11-00090] Kovacs Kristof, Conway Andrew R. A. (2016). Process overlap theory: A unified account of the general factor of intelligence. Psychological Inquiry.

[B33-jintelligence-11-00090] Liu Yan, Salvendy Gavriel (2009). Effects of measurement errors on psychometric measurements in ergonomics studies: Implications for correlations, ANOVA, linear regression, factor analysis, and linear discriminant analysis. Ergonomics.

[B34-jintelligence-11-00090] Luciana Monica, Bjork James M., Nagel Bonnie J., Barch Deanna M., Gonzalez Raul, Nixon Sara Jo, Banich Marie T. (2018). Adolescent neurocognitive development and impacts of substance use: Overview of the Adolescent Brain Cognitive Development (ABCD) baseline neurocognition battery. Developmental Cognitive Neuroscience.

[B35-jintelligence-11-00090] Mazefsky Carla A., Anderson Ranita, Conner Caitlin M., Minshew Nancy (2011). Child Behavior Checklist scores for school-aged children with autism: Preliminary evidence of patterns suggesting the need for referral. Journal of Psychopathology and Behavioral Assessment.

[B36-jintelligence-11-00090] Miller Adam Bryant, Sheridan Margaret A., Hanson Jamie L., McLaughlin Katie A., Bates John E., Lansford Jennifer E., Pettit Gregory S., Dodge Kenneth A. (2018). Dimensions of deprivation and threat, psychopathology, and potential mediators: A multi-year longitudinal analysis. Journal of Abnormal Psychology.

[B37-jintelligence-11-00090] Mills Kathryn L., Goddings Anne-Lise, Blakemore Sarah-Jayne (2014). Drama in the teenage brain. Frontiers for Young Minds: Neuroscience.

[B38-jintelligence-11-00090] Miyake Akira, Friedman Naomi P. (2012). The nature and organization of individual differences in executive functions: Four general conclusions. Current Directions in Psychological Science.

[B39-jintelligence-11-00090] Miyake Akira, Friedman Naomi P., Emerson Michael J., Witzki Alexander H., Howerter Amy, Wager Tor D. (2000). The unity and diversity of executive functions and their contributions to complex ‘frontal lobe’ tasks: A latent variable analysis. Cognitive Psychology.

[B40-jintelligence-11-00090] Moore Dawn Michele (2023). Open Science Framework. https://osf.io/nam56/.

[B41-jintelligence-11-00090] Mungas Dan, Widaman Keith, Zelazo Philip David, Tulsky David, Heaton Robert K., Slotkin Jerry, Blitz David L., Gershon Richard C. (2013). VII. NIH Toolbox Cognition Battery (CB): Factor structure for 3 to 15 year olds. Monographs of the Society for Research in Child Development.

[B42-jintelligence-11-00090] Mungas Dan, Heaton Robert, Tulsky David, Zelazo Philip David, Slotkin Jerry, Blitz David, Lai Jin-Shei, Gershon Richard (2014). Factor structure, convergent validity, and discriminant validity of the NIH Toolbox Cognitive Health Battery (NIHTB-CHB) in adults. Journal of the International Neuropsychological Society.

[B43-jintelligence-11-00090] National Institutes of Health (NIH), Northwestern University (2021). NIH Toolbox Scoring and Interpretation Guide for the iPad. https://nihtoolbox.my.salesforce.com/sfc/p/#2E000001H4ee/a/2E000000UZ7R/L8Da2nlj_FBx1LyO25ABnlyCy9HNYWMtG.uBNIbgLF0.

[B44-jintelligence-11-00090] Nelson J. Ron, Benner Gregory J., Cheney Douglas (2005). An investigation of the language skills of students with emotional disturbance served in public school settings. The Journal of Special Education.

[B45-jintelligence-11-00090] Nelson Timothy D., Kidwell Katherine M., Nelson Jennifer Mize, Tomaso Cara C., Hankey Maren, Espy Kimberly Andrews (2018). Preschool executive control and internalizing symptoms in elementary school. Journal of Abnormal Child Psychology.

[B46-jintelligence-11-00090] Neumann Denise, Peterson Elizabeth R., Underwood Lisa, Morton Susan M. B., Waldie Karen E. (2021). Exploring the factor structure of the NIH Toolbox Cognition Battery in a large sample of 8-year-old children in Aotearoa New Zealand. Journal of the International Neuropsychological Society.

[B47-jintelligence-11-00090] Nixon Sara Jo (1995). Assessing cognitive impairment. Alcohol Health and Research World.

[B48-jintelligence-11-00090] Osborne Jason W. (2015). What is rotating in exploratory factor analysis?. Practical Assessment, Research & Evaluation.

[B49-jintelligence-11-00090] Patwardhan Irina, Nelson Timothy D., McClelland Megan M., Mason W. Alex (2021). Childhood cognitive flexibility and externalizing and internalizing behavior problems: Examination of prospective bidirectional associations. Research on Child and Adolescent Psychopathology.

[B50-jintelligence-11-00090] Peter-Hagene Liana C., Burke Kelly C., Bottoms Bette L., Carris Kari Nysse, Conway Andrew R. A. (2019). Children’s eyewitness lineup accuracy one year later: The role of social support and working memory capacity. International Journal on Child Maltreatment: Research, Policy and Practice.

[B51-jintelligence-11-00090] Rey-Mermet Alodie, Gade Miriam, Souza Alessandra S., von Bastian Claudia C., Oberauer Klaus (2019). Is executive control related to working memory capacity and fluid intelligence?. Journal of Experimental Psychology: General.

[B52-jintelligence-11-00090] Salthouse Timothy A., Babcock Renée L., Shaw Raymond J. (1991). Effects of adult age on structural and operational capacities in working memory. Psychology and Aging.

[B53-jintelligence-11-00090] Schmidt Frank L., Hunter John E. (1996). Measurement error in psychological research: Lessons from 26 research scenarios. Psychological Methods.

[B54-jintelligence-11-00090] Schneider W. Joel, McGrew Kevin S., Flanagan Dawn P., McDonough Erin M. (2012). The Cattell-Horn-Carroll Model of Intelligence. Contemporary Intellectual Assessment: Theories, Tests, and Issues.

[B55-jintelligence-11-00090] Schoemaker Kim, Mulder Hanna, Deković Maja, Matthys Walter (2013). Executive functions in preschool children with externalizing behavior problems: A meta-analysis. Journal of Abnormal Child Psychology.

[B56-jintelligence-11-00090] Schreiber James B., Nora Amaury, Stage Frances K., Barlow Elizabeth A., King Jamie (2006). Reporting structural equation modeling and confirmatory factor analysis results: A review. The Journal of Educational Research.

[B57-jintelligence-11-00090] Spearman Charles E. (1904). ‘General intelligence,’ objectively determined and measured. The American Journal of Psychology.

[B58-jintelligence-11-00090] Strauss Esther, Sherman Elisabeth M. S., Spreen Otfried (2006). A Compendium of Neuropsychological Tests: Administration, Norms, and Commentary.

[B59-jintelligence-11-00090] Thompson Wesley K., Barch Deanna M., Bjork James M., Gonzalez Raul, Nagel Bonnie J., Nixon Sara Jo, Luciana Monica (2019). The structure of cognition in 9 and 10 year-old children and associations with problem behaviors: Findings from the ABCD Study’s baseline neurocognitive battery. Developmental Cognitive Neuroscience.

[B60-jintelligence-11-00090] Tuholski Stephen W., Engle Randall W., Baylis Gordon C. (2001). Individual differences in working memory capacity and enumeration. Memory & Cognition.

[B61-jintelligence-11-00090] Unsworth Nash, Engle Randall W. (2007). The nature of individual differences in working memory capacity: Active maintenance in primary memory and controlled search from secondary memory. Psychological Review.

[B62-jintelligence-11-00090] Wang Yiji, Zhou Xiaohui (2019). Longitudinal relations between executive function and internalizing problems in grade school: The role of peer difficulty and academic performance. Developmental Psychology.

[B63-jintelligence-11-00090] Wiebe Sandra A., Sheffield Tiffany, Nelson Jennifer Mize, Clark Caron A. C., Chevalier Nicolas, Espy Kimberly Andrews (2011). The structure of executive function in 3-year-olds. Journal of Experimental Child Psychology.

[B64-jintelligence-11-00090] Woltering Steven, Lishak Victoria, Hodgson Nick, Granic Isabela, Zelazo Philip David (2016). Executive function in children with externalizing and comorbid internalizing behavior problems. Journal of Child Psychology and Psychiatry.

[B65-jintelligence-11-00090] Zelazo Philip David (2006). The Dimensional Change Card Sort (DCCS): A method of assessing executive function in children. Nature Protocols.

[B66-jintelligence-11-00090] Zelazo Philip David (2015). Executive function: Reflection, iterative reprocessing, complexity, and the developing brain. Developmental Review.

